# Neuronal Models for Studying Tau Pathology

**DOI:** 10.4061/2010/528474

**Published:** 2010-07-19

**Authors:** Thorsten Koechling, Filip Lim, Felix Hernandez, Jesus Avila

**Affiliations:** ^1^Centro de Biología Molecular “Severo Ochoa” (C.S.I.C.-U.A.M.), Facultad de Ciencias, Universidad Autónoma de Madrid, 28049 Madrid, Spain; ^2^CIBERNED, Centro de Investigación Biomédica en Red de Enfermedades Neurodegenerativas, 28031 Madrid, Spain; ^3^Departamento de Biología Molecular, Universidad Autónoma de Madrid, 28049 Madrid, Spain

## Abstract

Alzheimer's disease (AD) is the most frequent neurodegenerative disorder leading to dementia in the aged human population. It is characterized by the presence of two main pathological hallmarks in the brain: senile plaques containing *β*-amyloid peptide and neurofibrillary tangles (NFTs), consisting of fibrillar polymers of abnormally phosphorylated tau protein. Both of these histological characteristics of the disease have been simulated in genetically modified animals, which today include numerous mouse, fish, worm, and fly models of AD. The objective of this review is to present some of the main animal models that exist for reproducing symptoms of the disorder and their advantages and shortcomings as suitable models of the pathological processes. Moreover, we will discuss the results and conclusions which have been drawn from the use of these models so far and their contribution to the development of therapeutic applications for AD.

## 1. Introduction: Alzheimer's Disease, Neuropathology, and Clinical Characteristics

Senile plaques and neurofibrillary tangles are the two main histopathological characteristics of Alzheimer's disease. They were described for the first time by Alois Alzheimer in 1907, who discovered both structures in the autopsy of a brain from a patient who had exhibited severe cognitive impairment and memory loss. Although these hallmarks of the disease were established as long as a hundred years ago, the illness was not fully recognized as such due to the social dismissal of dementia as a normal part of the human ageing process [1]. From the late 1970s onwards however, extensive neurobiological research has been in progress to understand the disease and to develop therapeutic approaches. 

Today it is widely accepted that the basis for AD is biological and that senile plaques and neurofibrillary tangles are responsible for the inception of the disorder and also that especially the number of NFTs is proportionally related to the severity of the accompanying symptoms, such as memory loss, confusion, and cognitive failure. Plaques and tangles can be found *post mortem* mainly in the hippocampus and cerebral cortex but also in other areas of the brain important for cognitive functioning.

Early and late onset AD as well as familial and sporadic AD are distinguished based on the time in life of the patient when symptoms first occur and the involvement of gene mutations or chromosomal aberrations that can be related to the disease, respectively. Nevertheless, in all cases the development of histopathological and behavioral symptoms is similar to indistinguishable. Several genetic factors have been described in relation with early and late onset familial AD, though their involvement is not* per se* essential for development of the disorder, given that only about 1% of all cases of Alzheimer's are familial. 

However, studying how these genetic influences may be able to induce the symptoms characteristic for AD could be a step towards understanding the mechanisms of the disease and lead scientists towards future clinical therapeutic approaches. Late onset familial AD may involve various susceptibility genes [2, 3]. The most studied is the APOE e4 allele of the gene coding for Apolipoprotein E. Though the underlying mechanism is still unknown, proof exists that this allele causes a shift in age of onset towards a younger age [4]. In early onset familial AD, mutations have been described in three genes involved in senile plaque formation, namely APP, PSEN1, and PSEN2 which encode the proteins Amyloid precursor protein, presenilin-1, and presenilin-2, respectively. The presenilins are integral membrane proteins that form part of the *γ*-secretase complex that, after *β*-secretase cleavage of the Amyloid precursor protein, generates the amyloid *β* (A*β*) fragment. Mutations in PSEN1 and PSEN2 are believed to possibly contribute to an increase in A*β*, especially the more neurotoxic form comprised of 42 amino acids, A*β*_42_. Mutated forms of human APP have been expressed in a variety of transgenic animal models to further the understanding of plaque formation.

Other contributing factors to the pathogenesis of AD are being addressed in various studies, and today lifestyle choices, adequate nutrition, psychological well being, and intellectual stimulation are proven to exert important influence on susceptibility to AD and other forms of dementia [5, 6]. The understanding and distribution of this information may not provide a cure but help to prevent the development or lower the severity of AD symptoms in many cases.

### 1.1. Senile Plaques

Senile plaques are formed via the aggregation of amyloid *β* outside neuronal cells. While A*β* is a naturally occurring 4 kDa polypeptide in the brain, it becomes neurotoxic in excess quantities or when it fails to be degraded so that polymerization occurs. Extracellular plaques developing in this manner act as synaptotoxins blocking the communication between neurons.

Plaques are formed mostly from A*β* derived from amyloid precursor protein (APP) which is an integral membrane protein type I of unknown function which occurs in different isoforms. The most common isoform which is expressed exclusively in neurons is APP695, comprised of 695 amino acids. APP contains various domains: a single transmembrane domain, an extramembranous N-terminal domain, and a cytoplasmic region at its C-terminus as well as a signal sequence. The neurotoxic effects of APP are believed to be mediated by proteolytic processing of APP which gives rise to A*β* [7]. *β*-secretase (BACE1, cleaving enzyme of the *β*-site of APP) liberates the amino terminal fragment of A*β* while the subsequent cleavage by *γ*-secretase of the resulting C-terminal fragment determines the overall length of the amyloid peptide, giving rise to A*β*_40_, and the less common but more neurotoxic form A*β*_42_. *α*-secretase cleavage of APP does not contribute to plaque formation due to the fact that this cleavage takes place inside the A*β* region of APP. A*β* is believed to be one of the principal factors which causes neurodegeneration in Alzheimer's disease by forming oligomeric aggregates leading to accumulation of these structures in the brain [8]. A*β*_42_ is considered to be more prone to aggregation and probably acts as a catalyst for the aggregation of A*β*_40_ [8]. 

### 1.2. Neurofibrillary Tangles

Neurofibrillary tangles (NFT) are the second histopathological hallmark found in brains affected by AD. These intraneuronal lesions consist principally of aberrant filaments, the principal proportion (95%) being paired helical filaments (PHFs), and the rest being straight filaments (SFs). PHFs are bundles made up of twisted filaments, which like SFs, are composed of aberrantly hyperphosphorylated tau protein [9, 10]. The diameter range of PHFs is 8–20 nm and they exhibit periodic repeats of 80 nm along their length while straight filaments do not show this periodicity and have a diameter of 15 nm [11]. PHFs are also the components of the neuropil threads, which appear independently of plaques and tangles and may be observed in an array of dystrophic neurites. Not all neuronal cell types appear to develop neurofibrillary tangles. In the cerebral cortex, all cells containing NFTs are pyramidal neurons, while in the subcortical nuclei the most affected cells are the ones with extremely long axons, consistent with the observation of thin long neurites being especially vulnerable to AD-related cytoskeletal changes [12].

NFTs also appear independently from the presence of senile plaques in other neurodegenerative disorders such as Pick's disease, Progressive supranuclear palsy [13], frontotemporal dementia and parkinsonism linked to chromosome 17 [14], meningioangiomatosis [15], or subacute sclerosing panencephalitis [16].

Under normal physiological conditions however, tau is a phosphoprotein that plays an important role in a variety of processes. Besides its crucial role in tubulin assembly and microtubule stabilization [17, 18], it is also important for the outgrowth of neurites from the cell body [19]. Recently, tau has also been observed to be involved in the migration of new neurons [20]. The authors detected phosphorylated tau protein in newly generated neurons in two well-known regions of adult neurogenesis, the subventricular zone associated with the lateral ventricles and the subgranular zone of the hippocampus. In these zones, phosphorylated tau was colocalized with doublecortin, a cytoskeletal protein that serves as a marker for neuronal migration, while tau knockout mice showed similar numbers of doublecortin-expressing cells but also a significant decrease in migration of these cells. The obtained results suggest a function for tau in the migration of newborn neurons in adult neurogenesis.

The modification of the tau protein by phosphorylation can alter the way it interacts with microtubules, as is the case with AD. There, hyperphosphorylation induces tau to dissociate from the microtubules which depolymerize while the concentration of soluble tau in the cells increases. This effect contributes to the assembly of filaments and the creation of PHFs from the soluble pool of tau. As neurofibrillary tangles are composed mainly of PHFs and their number in the brain has been described to be proportional to the severity of symptoms of dementia, phosphorylation of tau appears to play a major role in the pathogenesis of AD. 

Given these observations, it would be of great interest to determine the enzymes that phosphorylate tau in the brain. One of them has been identified as the kinase GSK-3. As an example, in a *Drosophila melanogaster* model, tau phosphorylation via its GSK-3 homologue Shaggy has been described to facilitate its aggregation to filamentous structures [21].

For an extensive explanation of tau and its role under physiological and pathological conditions, see the review published by our group [22].

An alternative interpretation of the role of phosphorylated tau protein has been promoted by the group of Mark A. Smith [23]. These authors question the concept that phospho-tau is inherently toxic because of its presence in neurofibrillary tangles and must therefore be a direct mediator of the disease. Instead, tau aggregation could be a response to the disease, and actually play the role of a protective shield against neurotoxic agents rather than leading to neurodegeneration. In support of this model Castellani and coworkers point out that NFTs, (a) are found in viable neuronal cells even in late stages of AD, (b) exist in the neuronal cytoplasm for decades, (c) can be observed, sometimes at high concentrations, in the brains of elderly persons who showed no signs of dementia throughout their lifetime, and (d) are present in neurons which contain normal amounts of structurally intact microtubules [24]. 

## 2. Animal Models for AD and Related Tauopathies

Several animal models of AD have been created in order to emulate specific features of the disease, such as its histopathological, biochemical, and behavioral characteristics. These models were designed to probe the pathological and biochemical changes that take place in affected organisms throughout the progression of the disorder and to test for possible therapeutic measures to be applied in the future in human patients.

The usefulness of an animal model depends on its capability of faithfully replicating the physiological processes that take part in the progress of the disease in human patients so that it leads to a better comprehension of the pathogenesis and finally allows developing an efficient therapeutic approach.

Since no other natural species spontaneously produce all of the histopathological, cognitive, and behavioral symptoms that characterize Alzheimer's, there was a need for the development of transgenic animal models or of dietary manipulation of test animals which would allow reproduction of the hallmarks of AD. From a practical viewpoint, it is of great importance to choose animals with a short lifespan and fast rates of ageing in order to be able to observe, in a reasonable time window, processes which take from 50 to 80 years in humans to appear and develop.

Each animal model has its limitations and advantages, as to date, none are able to express the whole set of characteristics needed to resemble full-blown AD pathology (Table 1). The contribution of each model to the understanding of this devastating disease, however, is immeasurable and has permitted the scientific community to establish an extensive base of knowledge on AD and the mechanics of the disorder. In the following, we will present the different animal models which have been established so far, discuss their characteristics, advantages, and pitfalls as well as take a look at the latest developments in this field of research.

### 2.1. Lower Eukaryote Models

Different invertebrate models have been created for studying Alzheimer's disease, the most commonly used organism being the fruit fly *Drosophila melanogaster*. Besides being easy to breed, manipulate, and genetically modify, *D. melanogaster* presents the advantage of having an extremely short development time spanning only 12 days from a fertilized egg to an adult fly, so the generation of large numbers of offspring is very easy. In one model, transgenic *Drosophila* expressed human wild type and R406 mutant tau [25]. Although overexpression of either tau form led to the premature death of the flies, symptoms of progressive neurodegeneration were more pronounced in the strain carrying the mutant gene. Intriguingly, the symptoms of neurodegeneration were not accompanied by the formation of neurofibrillary tangles. However, when flies which expressed wild type tau were induced to also overexpress the *Drosophila* GSK-3 homologue shaggy, neurofibrillary lesions could be observed. These findings indicate that higher levels of tau phosphorylation cause its aggregation into filaments and thus indicate an important role for GSK-3 in contributing to the formation of neurofibrillary tangles in AD. Drosophila was also used to investigate the neurotoxicity of human wild type and mutant tau in the early stages of embryonic development [26]. These experiments revealed that in spite of the pan-neuronal expression of tau in these transgenic lines, different anatomical patterns of toxicity in the CNS of these flies could be observed depending on whether the expressed isoform was wild type or mutant. While human WT tau was observed to be hyperphosphorylated at specific sites and caused severe abnormalities in the development of the mushroom body (MB) of the animals, the FTDP17 mutant isoform led to significantly less severe aberrations in MB development. The data from these experiments in *D. melanogaster* suggest that not only high levels of phosphorylation are required to mediate tau toxicity but also that modification has to take place at specific phosphorylation sites, and that tau toxicity is cell type-dependent. Another study points in the same direction, coming to the conclusion that specific phosphorylations at various sites modulate tau toxicity in a synergistic manner [27].

Modeling of A*β* plaque formation in *D. melanogaster* has been another objective of animal studies. The transgenic expression of human WT or mutant APP led to neuronal death in the brain already at the larval stage of development [28]. The severity of the symptoms was proportional to the concentration of A*β* and the C-terminal fragment of APP. Clues exist that the different forms of A*β* as soluble, oligomeric, and insoluble plaque deposits exhibit different toxicities towards cells. This fly model together with the recently generated specific antibodies against the different types of A*β*-aggregates [29, 30] could be used to address this question. *D. melanogaster* has also been successfully employed in the study of the influence of oxidative stress on the pathology of Alzheimer's disease. Lowering the antioxidant defenses of animals which expressed R406 mutant human tau increased tau toxicity while the antioxidant *α*-tocopherol (vitamin E) was able to alleviate the effects of tau toxicity [31].

Another application of the fruit fly with direct clinical relevance is that of a model organism for first round drug screening in AD. Given its extremely short development period, being an organism which is cheap and easy to manipulate even in large numbers and the low ethical restrictions when working with flies, *D. melanogaster* presents an overall advantageous choice. In a very recent study, a *Drosophila* model was described using the fly's notal bristles as a tool for assessing tau-induced toxicity [32]. The authors propose the use of this model for the screening of possible drugs for use in AD therapy. While the fruit fly presents a number of advantages as a suitable animal model for AD, there are also certain pitfalls. One major restriction when trying to extrapolate experimental data gained with *D. melanogaster* is its significantly different brain structure from humans, as it does not possess a hippocampus for example. Due to its small size the *Drosophila* brain is also difficult to analyse using stains for identification of distinct regional expression of histopathological markers. Last, memory impairment and cognitive deficits in such a phylogenetically distant organism are difficult to extrapolate to compare with human conditions.

To widen the understanding of the cellular mechanisms involved in AD and to aid in the search for pharmacological compounds that could be in the benefit of therapeutic interventions for tauopathies, other lower organisms are being employed as models for the disease. Two of the most widely used animal models at the time are the roundworm *Caenorhabditis elegans* and the zebrafish (*Danio rerio*). The advances that these models provided have been summarized in recent literature reviews [33, 34] for *C. elegans* and [35, 36] for *D. rerio,* respectively. *C. elegans* is a small organism with a short life span that allows for high throughput manipulation and drug screening applications, while the zebrafish, another well-suited organism for studying neurodegeneration, presents the advantage of being completely transparent in its larval development state, allowing continuous in vivo observation of processes taking place in its exposed nervous system. Paquet and coworkers [37] generated a fluorescently labeled tau transgenic zebrafish model. Using this animal, the authors were able for the first time to trace directly neurodegenerative processes taking place in the fluorescently stained larvae and to visualize the resulting neuronal cell death via time lapse microscopy in vivo. In addition, they employed this novel model as a tool for drug screening of GSK-3 inhibitors and have validated one promising compound termed AR-534.

### 2.2. Mouse Models

Choosing a neuroanatomy which more closely resembles the human brain, several mouse models of AD and other tauopathies have been developed in the last fifteen years. Earlier models fell into two main groups, according to the AD hallmark lesion (amyloid plaques or neurofibrillary tangles) mimicked in each model, but more recent approaches have generated models exhibiting both features simultaneously.

#### 2.2.1. APP/A*β* Modeling

Several transgenic mouse lines have been generated which express one of the mutant forms of APP. Most of these transgenic lines exhibited senile plaques as A*β* depositions and also the characteristic behavioral deficits reminiscent of AD in animal models. The first of the transgenic animals expressed the human mutant V717F APP form driven by the platelet-derived growth factor (PDGF) mini promoter [38]. A*β* plaque formation was observed in the test animals as well as memory impairment, especially related to spatial learning. When compared to wild type mice, the transgenic animals showed a significantly more severe decline in memory as assessed by a modification of the Morris water maze experiment. Another model expressed a different mutant APP form, the APP^SW^ (Swedish) double mutation inserted into a hamster prion protein (PrP) cosmid vector [39]. The animals presented memory and spatial learning deficits at 9 months of age. *β*-amyloid concentrations increased fivefold in A*β*_40_ and 14-fold in A*β*_42_, and the deposits could be stained with Congo red especially in the cortical and limbic regions of the brain. In a third model that overexpressed APP sevenfold, amyloid plaques appeared as soon as at six months of age in the tested mice. In contrast to the other two cited studies, these mice showed significant neuronal cell death besides the plaque deposits, specifically of pyramidal neurons in the CA1 region of the hippocampus, with the plaques seemingly affecting cell integrity in the adjacent neurons. Several experiments in transgenic mice have shown that amyloid plaque formation can promote tau pathology [40, 41]. When crossing human APP transgenic mice with tau expressing strains, or administering A*β*_42_ intracerebrally, tau phosphorylation was enhanced and NFT depositions increased in concentration. Interestingly however, when hAPP mice were crossed with tau knockout animals, no memory or learning deficits could be detected in spite of the massive deposition of plaques in these mice [42]. These findings underscore the importance of the presence of tau protein for the induction of the disease as in this animal model amyloid-mediated toxicity was nullified by the absence of tau. The level of neurotoxicity exerted by A*β* also depends on its length as was revealed in a transgenic mouse study carried out by McGowan and coworkers [43], as transgenic mice which express only A*β*_40_ did not develop any senile plaques while A*β*_42_-expressing animals did.

A different mechanism of proteolytic modification of the Amyloid precursor protein is the cleavage at aspartate residue 664 (D664) by caspases. Previous studies suggested that this cleavage, alternative to the described catalytic mechanisms involving secretases, could play a key role in the pathogenesis of AD [44, 45]. To address this question, Harris and coworkers used two transgenic mouse lines carrying the APP gene with (B254) and without (J20) the caspase-specific cleavage site and studied the possible implication of the resulting products, the C31 and Jcasp fragments, in AD [46]. Although these products had been described before to cause cell death in vitro, in these in vivo experiments, histological and behavioral assessment of the test animals did not reveal significant differences between the B254 and J20 mice. The authors came to the conclusion that caspase cleavage of APP does not play a critical role in the generation of AD-related abnormalities in these transgenic mice, and that therefore the D664 cleavage site of APP would not be a suitable target for the development of therapeutic interventions.

Recently, the differential toxicity of soluble A*β* oligomers and fibrillary A*β* plaques has been discussed [47]. In a study with mice, animals which overexpressed the “Arctic” mutation were compared to mice overexpressing wildtype A*β* [48]. While the mutant A*β* (A*β*E22G) led to the marked formation of amyloid plaques, it also lowered the concentration of a specific nonfibrillar A*β*-assembly (Aβ*56). Remarkably, both strains showed similar behavioral and neuronal deterioration when normalized for Aβ*56 levels, while the number of plaques was very different. The authors of the study concluded that Aβ*56 concentrations are a more suitable marker for AD-related functional deficits than the amount of A*β* deposited in the form of plaques. Therapeutic approaches that lead to the breakdown of fibrillary A*β* but possibly increase the levels of soluble oligomers could therefore be counterproductive. 

In view of these data, therapeutic interventions which block the production of A*β* monomers and soluble oligomers should be explored. This could be achieved by inhibiting the secretases involved in APP processing. These enzymes however are involved in other physiological processes as well, and it may therefore be detrimental to indiscriminately reduce their activity. Recent strategies avoid this problem, as in the case of some nonsteroidal anti-inflammatory drugs (NSAIDs) which do not alter secretase activity but alter its cleavage site specificity resulting in the generation of the less toxic 38 residue A*β* instead of the highly amyloidogenic A*β*42 [49]. The introduction of immunotherapeutic treatments could also lead to the specific elimination of A*β* oligomers and might prevent their formation. Positive effects of this approach have been described in a transgenic mouse model [50] and were also assessed in humans in a small cohort of AD patients [51]. 

#### 2.2.2. NFT Modeling

Transgenic mice carrying cDNAs which encode either the largest or the smallest human tau isoform have been generated. Later, mouse models which express the mutant isoform of tau found in FTDP-17 patients were also developed, as well as mice that model the disease via the overexpression of certain kinases which play a key role in the hyperphosphorylation of tau in AD and related disorders. 

The first transgenic mouse which expressed the longest tau isoform under control of the human Thy-1 promoter showed tau phosphorylation at sites which are usually found to be modified in PHFs and presented localization of human tau in neuronal soma, axons, and dendrites. These mice exhibit modest expression of human tau protein (approximately 10% of total tau in the animals) and did not exhibit neurofibrillary tangles [52]. However, the histopathology observed in these animals reflects an early stage of AD prior to the formation of neurofibrillary tangles, in which hyperphosphorylated forms of tau are localized in the soma and dendrites of neuronal cells. In another mouse model in which the shortest human tau isoform was overexpressed [53] under the murine HMG CoA reductase promoter, transgenic tau could be detected in the somatodendritic compartment, although again no NFT formation was observed. Subsequent transgenic mouse models were designed using stronger promoters, resulting in increased expression of human tau in these animals until eventually brain tau aggregates could be detected, although the formation of NFTs still remained elusive [54, 55]. These models showed not only AD-like symptoms in brain cells but also exhibited spheroidal tau aggregates in the spinal cord resulting in motor symptoms in the animals characteristic more of amyotrophy [56]. While transgenic mice with excessive overexpression of human tau are not viable, lines that overexpress tau less than tenfold have been generated and tau inclusions have been observed in cortical, brain stem, and spinal cord neurons, accompanied by other symptoms such as axon degeneration, decrease of microtubules, and motor deficits. Staining of the inclusions with the AD pathology-specific dyes Congo red and Thioflavin S revealed increasing insolubility of the aggregates over time and NFT-like inclusions could be detected in old animals (18 to 20 months) [57]. Other models with the tau gene under the control of the PrP promoter led to the expression of high levels in certain types of neurons and glial cells. Here, fibrillary structures could be detected in glial cells (oligodendrocytes) as well as neurons [40]. The phenotype however was still not severe. In a mouse model expressing three isoforms of human tau simultaneously, structures were observed which were similar to the astrocytic plaques that characteristically appear in the gray matter in cases of Corticobasal Degeneration (CBD), although neuronal cells were not equally affected, as they did not show any fibrillary lesions [58].

While to this day no mutations have been found in the tau-encoding MAPT gene in AD patients, molecular analysis of another tauopathy, frontotemporal dementia with parkinsonism linked to chromosome 17 (FTDP-17), has revealed characteristic mutations in this gene. Some of them like the P301L or the R406W mutations have been observed to lower tau's potential to promote microtubule stability [59]. In mouse models expressing a human tau isoform containing the P301L mutation [60], reviewed in [61], it was found that this mutation reduces the affinity of tau for microtubules. In one study, the mice showed NFTs in the brain as well as in the spinal cord alongside with a substantial reduction in the number of motor neurons [60]. Furthermore, the animals developed severe behavioral deficits which emulate neurological symptoms of the disease in humans. In another study also employing mice overexpressing human tau with the P301L mutation [40], short tau filaments were isolated from the brains of the test animals. Interestingly, one study revealed that when the expression of mutant P301L tau was suppressed after a period of overexpression, the behavioral symptoms in these mice could be reversed, while the insoluble NFTs were not removed but continued to accumulate. This result hints at the possibility that soluble tau rather than its fibrillary deposits is the cause of neuronal cell death in Alzheimer's disease [62]. A triple transgenic mouse model expressing the mutant transgenes PS1 (M146V), APP^SW^, and P301L tau was generated by the group of LaFerla to examine the interactions between beta-amyloid, neuronal dysfunction and neurofibrillary tangles [63]. These 3xTg-AD mice developed both of the classical hallmarks of Alzheimer's in human patients, senile plaques and neurofibrillary tangles, and furthermore, synaptic plasticity and neuronal long-term potentiation were impaired in these animals in a manner which could be related to A*β* formation.

In 2002, a transgenic mouse was created that expressed tau bearing the P301S mutation. This mutation is responsible in humans for an early onset of the pathological signs of FTDP-17 [64]. In this model, numerous PHFs consisting of tau filaments could be detected in the brains and spinal cords of the animals. Another observation was that motor neurons were the cell type where the highest concentrations of tau could be detected. Consistent with the findings in human patients where the P301S mutation is related with an earlier onset of FTDP-17 as compared to the P301L mutation, neuronal cell death was highly elevated (49%) in mice which overexpressed human P301S tau. Colocalization of hyperphosphorylated tau deposits and MAP kinases hinted at the possible implication of these enzymes in tau modification. In another study which compared the development of neurodegenerative signs in P301S mutant transgenic and wild type mice, neurofibrillary lesions could be detected in the transgenic animals at the age of 9 to 12 months, accompanied by the massive loss of hippocampal and cortical neurons [65]. Interestingly, synapse loss in the hippocampus and deficits in synaptic function could be observed long before the formation of NFTs, at about three months of age. Given that early microglial activation was also observed and that tau pathology could to some extent be reverted by administration of the immunosuppressive drug FK506, the authors of the study established a link between neuroinflammation and the early stages in the development of tauopathies.

The implication of an improperly controlled cell cycle in neurodegenerative diseases has also been described in the literature [66]. In degenerating neurons elevated concentrations of proteins associated with the cell cycle such as cyclins, cyclin-dependent kinases, and the products of proto-oncogenes such as c-myc, can often be detected. Two studies investigate the effects of cell cycle reentry mediated by transduction with c-myc, using an in vitro cell culture model [67] as well as a transgenic mouse model [68]. Induced cell cycle reentry led to the death of neurons, gliosis, and cognitive impairment in the mouse model, thus indicating that this loss of control in mature, postmitotic neurons may contribute to the pathogenesis of diseases like Alzheimer's. Interestingly, forced cell cycle re-entry led to the phosphorylation of tau and to the formation of tangle-like structures in the in vitro model, providing further evidence for a possible causal connection between the cell cycle and AD. 

Another interesting study addresses the possible contribution of insulin to the pathogenesis of AD [69]. As insulin has been described to be involved in the metabolism of both A*β* and tau, the researcher studied the possible effect of artificially induced Diabetes mellitus (DM) on the brain cells of pR5 transgenic mice which expressed the P301L human mutant tau and produced neurofibrillary tangles. After drug induced-insulin depletion, tau phosphorylation increased in both wild type controls and transgenic mice, though remarkably only the transgenic animals produced massive deposits of nonsoluble hyperphosphorylated tau protein. The authors came to the conclusion that DM is capable of triggering an earlier onset of a preexisting tau pathology in susceptible animals and that diabetes might cause an abnormal phosphorylation of tau via the elevation of glucose levels. Given the high rates of comorbidity of AD and DM in the human population, this line of research deserves further investigation as it might unveil new insights on the pathogenesis of AD.

In a complex yet very elegant survey, a genome wide search strategy employing lentivectors led to the identification of a retroposed gene in mouse [70]. This Rps23r1 gene normally encodes for the ribosomal protein S23 but in the retroposed form is transcribed in the reversed sense, expressing a functional protein which is localized integrated in cell membranes of the cerebral cortex and hippocampus. Intriguingly, overexpression of Rps23r1 reduced levels of A*β*, GSK-3 activity, and tau phosphorylation. The proposed mechanism of this effect consists of an initial interaction with adenylate cyclases upregulating cellular cAMP levels, which in turn activates protein kinase A (PKA). This activation results in the inhibition of GSK-3, a kinase that is involved in tau phosphorylation and A*β* generation.

Given that these aberrant phosphorylations of tau play a key role in the development of Alzheimer's disease, another important strategic approach is to identify the phosphorylation sites that are connected to the formation of aggregates and to identify the responsible enzymes and pathways for these tau modifying reactions, namely, the kinases and phosphatases.

The first generation of transgenic mice expressing GSK-3*β*, a kinase involved in many physiological pathways, was generated using both ubiquitous or CNS-specific promoters [71]. In all cases however, though slight increases in the phosphorylation levels of tau could be observed, no overexpression of the enzyme took place. This is probably due to the narrow window of concentrations in which GSK-3 can be expressed in cells, below and above which the lack or excess of GSK-3 activity proves lethal. 

Considering the narrow concentration range of GSK-3 that permits cell viability, a model was created with GSK-3 gene expression adjustable by means of a conditional tetracycline regulated system under the control of the CaM kinase II *α*-promoter [72]. The advantage of animal models employing the tetracycline regulation system lies in the possibility to carry out reversible studies [73]. In this mouse model, GSK-3*β* overexpression was confined to a particular set of cortical and hippocampal neurons. The overexpression of the kinase led to hyperphosphorylation of tau as detected by specific antibodies and showed how tau phosphorylation lowers its affinity for microtubule binding. Behavioral deficits related to Alzheimer's disease however, could be observed in this animal model by applying the Morris water maze test [74]. In spite of these findings, the correlated deposition of insoluble tau neurofibrillary tangles could not be observed. The shutdown of GSK-3 overexpression in turn leads to normal GSK-3 activity, normal phospho-tau levels, diminished neuronal death, and the suppression of the cognitive deficits. These findings further support the potential of GSK-3 inhibitors in the treatment of AD [75].

To further study the involvement of GSK-3, transgenic mice that overexpress GSK-3*β* were crossed with FTDP-17 mutant tau mice [75]. This AD animal model, termed GSK-3/VLW, shows tau hyperphosphorylation in CA1 hippocampal neurons, the region where the expression patterns of both transgenes overlap. Tau filaments with a PHF-like structure were found in GSK-3/VLW mice but not in single transgenic mice expressing either GSK-3*β* or FTDP-17 tau alone. PHF-like filament formation in GSK-3/VLW mice was accompanied by thioflavin-S staining, indicating the presence of senile plaques. All these data suggest that there is a synergistic contribution of both types of tau modification, hyperphosphorylation and missense mutations, to induce aberrant tau aggregation. 

The same animal model has been utilized to study the possible effects of lithium, a GSK-3 inhibitor used for treating affective disorders with well-documented effects in humans [76]. Two questions were addressed: first, whether chronic lithium treatment is able to prevent the formation of aberrant tau aggregates (formed by overexpression of FTDP-17 tau and GSK-3*β*); and second, whether lithium can revert already formed tau aggregates and NFTs to achieve their clearance in aged animals. The results indicated that lithium is capable of preventing the development of tau pathology when administered early in disease progression. On the other hand, if lithium administration is initiated at late stages, tau hyperphosphorylation is reduced but tau aggregation cannot be reversed. The data supports studies describing novel GSK-3 inhibitors as new pharmacological treatments of this kind of neurodegenerative disorders, as reviewed byAvilaand Hernández [77]. 

A second transgenic animal expressing a constitutively active form of the kinase, GSK-3*β*(S9A), has been cross-bred with transgenic mice that overexpress the longest human tau isoform [55]. The number of axonal enlargements present and the motor impairment typical for these tau transgenic animals were reduced in the double transgenic mice [78]. Thus, taking into account all these data it seems that GSK-3 could have different functions in different neurons and in different regions of the brain, while the hippocampal dentate gyrus seems to be more susceptible to degeneration in transgenic mice overexpressing GSK-3*β* [75]. A recent publication examines the possible role of GSK-3 in tau phosphorylation in the dentate gyrus [79]. Previously generated transgenic mice which overexpress GSK-3 in the dentate gyrus were crossed with a tau knockout strain in order to verify if GSK-3 mediated phosphorylation of tau is the cause of neurodegeneration in the dentate gyrus. When compared to tau-expressing control animals, the authors observed that the signs of neurodegenerative damage were significantly attenuated in the absence of tau. Therefore, hyperphosphorylation of tau is proposed to be a causal factor of the histopathological lesions found in the animals.

Very recently, the role of caspases in AD was reevaluated in a study by Calignon and coworkers [80]. Using the transgenic Tg4510 mouse strain, the researchers observed that tangle free cells which showed caspase activation formed NFTs within 24 hours. Furthermore, administration of wild type tau into wild type animals led to caspase activation and eventually to tangle formation. The data from this study suggest that caspase activation of tau could be a direct cause of tangle formation, and as has been pointed out in various recent articles, that the AD typical neurofibrillary deposits are the final histological outcome of a neurodegenerative process, rather than the cause of it.

## 3. Cell Culture Models

While research models in animals allow studies in the whole organism within a reasonable time, there also exists a need for the possibility to investigate disease-related processes in human cells. Live human neuronal cells however are difficult to access for study. Therefore, recently cell culture-based approaches are being developed, relying on the derivation and propagation of neuronal cells from different types of human tissue. 

One widely employed model for neuronal cells in culture is the human neuroblastoma line SHSY5Y, which was used to elucidate the role of leptin in Alzheimer's disease. Leptin has recently been proposed as another factor related to the susceptibility of contracting AD. It is an endocrine hormone with implications in food intake regulation as well as processes of learning and memory, for example, in long-term potentiation (LTP) [81]. Epidemiological data from human populations suggest a significantly lower risk of developing AD for people with higher leptin blood levels [82]. The mechanism of action is probably the same as one described for the physiologically closely related peptide insulin. Experiments with differentiated SHSY5Y human neuroblastoma cells indicate that both compounds are capable of reducing the level of tau phosphorylation via inactivation of GSK3-*β* [83]. Another study revealed a possible protective effect of leptin on neuronal cells against *β*-amyloid toxicity [84]. In as SHSY5Y cell culture model as well as in a transgenic mouse model, leptin was found to reduce toxic A*β* levels. Leptin reduces the activity of *β*-secretase, one of the enzymes that cleaves APP, which could probably be the underlying mechanism of the A*β* reducing effect of leptin.

Recently, the applicability of cell culture models has been vastly improved by the possibility of deriving patient-specific cell lines that can be propagated in vitro. Patient-specific fibroblasts for example, can be reprogrammed to induced pluripotent stem (iPS) cells and then differentiated into neuronal cell types similar to those found in the hippocampus or cerebral cortex. This allows the generation of cell models that reproduce certain disease-specific features in vitro, without ethical concerns and which are easily accessible for analysis and manipulation [85]. 

The groundbreaking work in iPS generation was conducted by the group of Yamanaka, who succeeded in creating pluripotent stem cells first by transducing mouse embryonic fibroblasts with a set of four genes encoding the factors Oct3/4, Sox2, c-Myc, and Klf4. When grown under culture conditions for embryonic stem (ES) cells, these iPS cells showed the morphological traits, growth potential, and immunomarkers of ES cells [86]. Later, the authors were able to generate iPS cells from adult human dermal fibroblasts using the same four factors [87]. Today, several cell lines specific for various neurodegenerative diseases have been generated with the objective of creating human in vitro models. Examples are Amyotrophic lateral sclerosis (ALS) [88], Parkinson disease (PD), and Huntington disease (HD), reviewed in [89]. In the case of ALS, Dimos and coworkers [88] were able to derive iPS cells from fibroblasts of an 82-year-old patient suffering from the disease and subsequently differentiate these iPS cells into motor neurons, the cell type affected by ALS. To date, the generation of Alzheimer's specific iPS cells has not been published, but their generation and differentiation to neuronal types involved in disease progression hopefully will be accomplished soon, offering a novel tool to study the pathological processes, such as tau phosphorylation and tangle formation, which so far have not been completely recapitulated in animal models. 

Another strategy is the use of fetal human neural stem cells (hNSC) to create cell models by differentiation of these cells to neuronal types of interest. We have established a method for the differentiation of hNSC to postmitotic neurons which express the neuronal markers tau and *β*III-tubulin. These neurons were derived from human fetal forebrain tissue [90] and are viable in cell culture for weeks. This model permits the study of effects of candidate therapeutic drugs on tau expression or phosphorylation state. Furthermore, these cells can be transduced with viral vectors to overexpress AD-related genes, such as those encoding tau or GSK-3 allowing the study of the consequent alterations, including the formation of tangles and plaques. 

Additionally, the effect of tau-overexpression on neural stem cells (in their undifferentiated state) is an interesting topic, as brains affected by AD show high levels of tau phosphorylation in regions, such as the hippocampus, where neural stem cells reside and are involved in adult neurogenesis. As research in neural stem cells and neurodifferentiation continues to make technological leaps, further contributions to Alzheimer research can be expected soon by means of creation of human cell models that imitate specific aspects of the disease.

## 4. Conclusion

In summary, a variety of animal models have been generated which reproduce either the A*β* or tau histopathological hallmark lesions of AD as well as transgenic animals which exhibit both features simultaneously. Some of these models reproduce the lesions as well as the related behavioral impairments, while suggesting that both plaques and tangles may have synergistic effects in driving the disorder rather than developing independently from each other. This observation is strengthened by the notion that the transgenic mouse models most successfully reproducing AD-like pathology are the ones which combine overexpression of tau and APP, like the 3xTg-AD mice [63]. The animal models shed light on mechanisms of progression of tauopathies and their application as drug screening systems pave the way for the development of future treatments of dementia. Due to the additional need for human models combined with the impossibility of conducting basic research in humans in vivo, the use of iPS cells and neural stem cell cultures derived from human fetal and adult tissue is being established as an alternative and complementary route. While this approach does not hold the advantages of conserving the integrity of the biological system under study, they do allow the study of molecular interaction and biochemical pathways in an all-human system, a point of high relevance due to the human specificity of many aspects of AD.

## Figures and Tables

**Table 1:** Overview of the most widely used models for studying Alzheimer's disease.

Model	Advantages	Drawbacks
*Mus musculus *(mouse)	(i) Highly evolved organism, brain anatomy, and metabolism close to humans, results often extrapolatable to humans (ii) Cognitive symptoms of neurodegeneration can be assessed (iii) Generation of conditional transgenic models possible	(i) Relatively long breeding/development time (ii) Ethical concerns (iii) Not suitable for drug screening (iv) Expensive

*Danio rerio *(zebrafish)	(i) Ex-utero development in transparent capsule allows for live imaging of neurodegenerative processes (ii) Short life cycle (iii) Genetic manipulation tools available	(i) Brain anatomy and genetic setup distinct from humans (ii) Behavior not sufficiently studied, difficult to evaluate cognitive deficits

*Caenorhabditis elegans *(nematode)	(i) Simple anatomy, (ii) Easy laboratory culture, short life cycle (iii) Genetic manipulation tools highly advanced	(i) Brain anatomy and genetics distinct from humans (ii) Cognitive/behavioral deficits difficult to assess

*Drosophila melanogaster * (fruit fly)	(i) Reference model for genetic studies (ii) Suitable for drug screening (iii) Short generation times, low maintenance costs (iv) genetic manipulation tools highly advanced	(i) Brain anatomy and genetics distinct from humans (ii) Cognitive/behavioral deficits difficult to assess

In vitro cell culture models	(i) Direct monitoring of parameters over time possible (ii) Extremely valuable for high-throughput screening for therapeutic drugs (iii) Easy handling, economic	(i) Organ structure not conserved, environmental cues/interaction with other organs are not taken into account (ii) Ethical considerations in the case of human embryonic/fetal tissue as source material
